# Molecular Detection of *Leishmania* in Phlebotomine Sand Flies (Diptera: Psychodidae) from a Cutaneous Leishmaniasis Focus at Xakriabá Indigenous Reserve, Brazil

**DOI:** 10.1371/journal.pone.0122038

**Published:** 2015-04-08

**Authors:** Felipe Dutra Rêgo, Jeronimo Marteleto Nunes Rugani, Paloma Helena Fernandes Shimabukuro, Gabriel Barbosa Tonelli, Patrícia Flávia Quaresma, Célia Maria Ferreira Gontijo

**Affiliations:** Grupo de Estudos em Leishmanioses, Centro de Pesquisas René Rachou, Fundação Oswaldo Cruz, Av. Augusto de Lima, 1715 Barro Preto, CEP 30190–002, Belo Horizonte, Minas Gerais, Brasil; Louisiana State University, UNITED STATES

## Abstract

Autochthonous cases of American cutaneous leishmaniasis (ACL) have been reported since 2001 in the Xakriabá Indigenous Reserve located in the municipality of São João das Missões in northern Minas Gerais state, Brazil. In order to study the presence of *Leishmania* DNA in phlebotomine sand flies, six entomological collections were carried out from July 2008 through July 2009, using 40 light traps placed in peridomicile areas of 20 randomly selected houses. From October 2011 through August 2012, another six collections were carried out with 20 light traps distributed among four trails (five traps per trail) selected for a previous study of wild and synanthropic hosts of *Leishmania*. A total of 4,760 phlebotomine specimens were collected belonging to ten genera and twenty-three species. Single female specimens or pools with up to ten specimens of the same locality, species and date, for *Leishmania* detection by molecular methods. Species identification of parasites was performed with ITS1 PCR-RFLP using *Hae*III enzyme and genetic sequencing for SSU rRNA target. The presence of *Leishmania* DNA was detected in eleven samples from peridomicile areas: *Lu*. *longipalpis* (two), *Nyssomyia intermedia* (four), *Lu*. *renei* (two), *Lu*. *ischnacantha*, *Micropygomyia goiana* and *Evandromyia lenti* (one pool of each specie). The presence of Leishmania DNA was detected in twelve samples from among the trails: *Martinsmyia minasensis* (six), *Ny*. *intermedia* (three), *Mi*. *peresi* (two) and *Ev*. *lenti* (one). The presence of *Leishmania infantum* DNA in *Lu*. *longipalpis* and *Leishmania braziliensis* DNA in *Ny*. *intermedia*support the epidemiological importance of these species of sand flies in the cycle of visceral and cutaneous leishmaniasis, respectively. The results also found other species associated with *Leishmania* DNA, such as *Mt*. *minasensis* and *Ev*. *lenti*, which may participate in a wild and/or synanthropic cycle of *Leishmania* transmission in the studied area.

## Introduction

Leishmaniases are endemic in many countries where they are considered an important public health problem. The etiological agents for leishmaniases are a variety of protozoan species of the genus *Leishmania* (Kinetoplastida: Trypanosomatidae) [1,2,3]. Infection occurs in a wide range of vertebrate hosts, including wild and domestic mammals such as rodents, canines, edentates and marsupials, and the vectors are hematophagous insects of the subfamily Phlebotominae (Diptera: Psychodidae) [1,4,5].

Expansion of the geographic distribution of leishmaniasis has been reported in several Brazilian states, including Minas Gerais (MG) where American cutaneous leishmaniasis (ACL) is endemic and widely distributed. Disease-endemic foci occur in the Rio Doce valley [6,7], the Jequitinhonha valley [8], urban centers in the northern region [9,10,11], and on the outskirts of the state capital Belo Horizonte [12,13].

Several species of sand flies (Phlebotaminae) have been associated with species of *Leishmania* in MG, such as: *Ny*. *intermedia* [14]; *Lu*. *longipalpis* [10,14], *Ny*. *whitmani* [14]; *Ny*. *neivai* [15], *Pintomyia fischeri*, *Pi*. *pessoai*, *Psychodopygus lloydi* and *Ps*. *hirsutus* [16,17,18]. In addition to these reports other phlebotomine species, whose epidemiological significance remains unclear, have also been found associated with species of *Leishmania*, such as: *Ev*. *cortellezzi* [19], *Ev*. *sallesi*, *Ev*. *termitophila* [14,15], *Ev*. *lenti*, *Pi*. *christenseni*, *Pi*. *monticola*, *Psathyromyia aragaoi* and *Ps*. *lutziana* [16].

In a previous study in the Xakriabá Indigenous Reserve (XIR) located in the northern region of MG, wild, synanthropic, and domestic hosts were found to be naturally infected by different species of *Leishmania* [20]. A recent study on the ecology of the phlebotomine sand fly fauna in the same area reported the presence of *Ny*. *intermedia* and *Lu*. *longipalpis* mainly in peridomicile areas, and *Martinsmyia minasensis* and *Lutzomyia cavernicola* mainly in wild area [21]. The aim of this study was to survey for *Leishmania* DNA among phlebotomine sand flies collected in a village located in the XIR where autochthonous cases of ACL have been reported since 2001.

## Materials and Methods

### Study area

**Fig 1:**
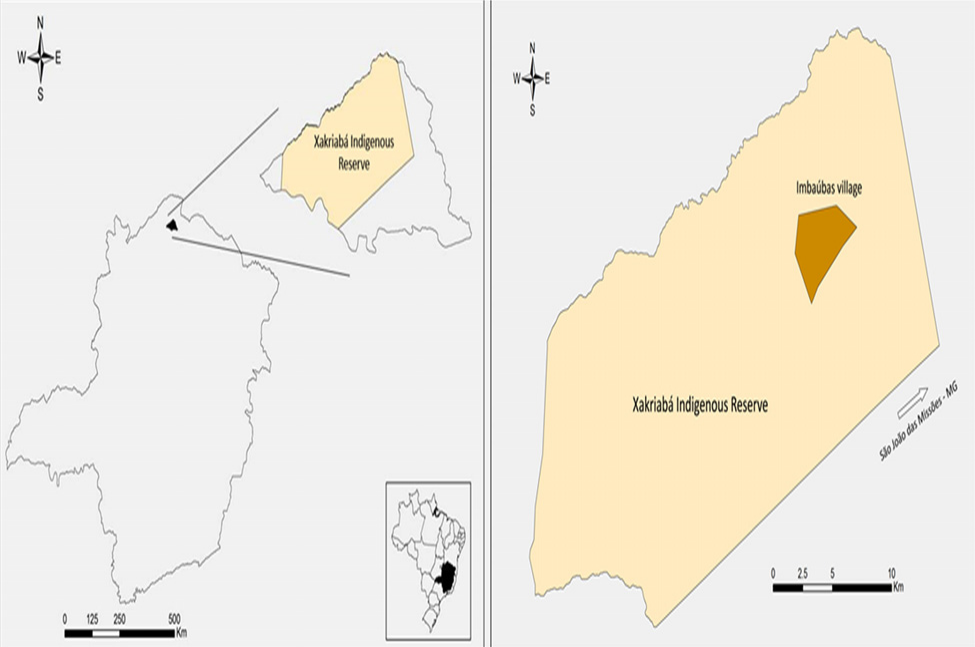
Location of study area. The location of the municipality of São João das Missões in northern Minas Gerais, Brazil. The native village of Imbaúbas located in the Xakriabá Indigenous Reserve, where the study was performed, is indicated.

The XIR is located in the municipality of São João das Missões (14°53′4.26“S 44°4′53.19”W) in the northern region of the state of Minas Gerais, Brazil (Fig 1). The indigenous reserve is located in a transition zone between cerrado and caatinga biomes and contains native species of both. This study was conducted in Imbaúbas, an indigenous village which has had both a high prevalence of ACL human cases and numerous wild, synanthropic and domestic *Leishmania* hosts [20]. Additionally, species incriminated as vectors of *Leishmania* have been recorded from the village by Rego et al. [21]. This study was conducted with the authorization of FUNAI (National Indian Foundation—Process Number: 2098/08).

### Sample collection and identification of phlebotomine sand flies

Sand flies used in the present study were originally sampled, collected and identified as published in [21]. The female specimens were pooled and prepared for DNA extraction as shown below.

### *DNA extraction and* Leishmania *identification*

Sand flies were tested with a minimum of one sand fly female species or pooled to a maximum of ten female specimens of the same species, locality, and date being placed in 1.5 mL tubes containing dimethylsulfoxide 6% (DMSO) and stored at -20°C until DNA extraction. DNA was extracted with Gentra Puregene (QIAGEN, USA) following instructions of the manufacturer. In order to control for potential contamination we included negative control groups (male sand flies) in the DNA extraction step and decontaminated instruments and working areas with DNAZap (Ambion Life Technologies, Inc.).

The extracted DNA from peridomicile areas and trails was screened for *Leishmania* by the amplification of a 300–350 bp fragment of the intergenic region of the *Leishmania* DNA (internal transcribed spacer 1—ITS1), using the primers LITSR: 5´ CTGGATCATTTTCCGATG 3´ and L5.8S: 5´ TGATACCACTTATCGCACTT 3 [22,23].

Tests for the presence of *Leishmania* DNA from peridomicile samples was also performed by nested PCR (LnPCR) with primers that were directed at the small subunit ribosomal ribonucleic acid gene—ssu rDNA [24,25,26]. The first amplification step was performed using R221 and R332 primers and the PCR products were then tested in a subsequent amplification step with R233 and R333 primers [25].

Positive controls for the PCR reactions included DNA extracted from promastigote forms of the following *Leishmania* strains: *Leishmania amazonensis* (IFLA/BR/67/PH8), *Le*. *braziliensis* (MHOM/BR/75/M2903), *Le*. *infantum chagasi* (MHOM/BR/74/PP75) and *Le*. *guyanensis* (MHOM/BR/75/M4147). Amplification products were subjected to electrophoresis in 2% agarose gel and stained with ethidium bromide (10mg/mL) with a 100 bp DNA Step Ladder provided as molecular weight size standard.

To identify species of *Leishmania*, the ITS1 PCR products (10–15 μL) were digested with *Hae*III (10U/μL) without prior purification using conditions recommended by the supplier (New England Biolabs, Ipswich, MA, USA). The restriction profiles were analyzed in 4% agarose gel, stained with ethidium bromide (10mg/mL), and compared with the *Leishmania* reference strains as previously indicated.

Each pool that tested positive by LnPCR and ITS1-PCR were purified using QIAquick PCR Purification kit (QIAGEN, USA) following the instructions of the manufacturer. The purified fragments were then sequenced using Big Dye Terminator v3.1 Cycle Sequencing Kit (Applied Biosystems, Foster City, CA, USA) in a final volume of 10 μL with 20ng of the purified PCR products and 3.3 pmol of the forward and reverse primers. The products were sequenced in duplicate for each primer (two forward and two reverse). Sequences were then generated by a ABI 3730xl DNA Analyzer, and the software Finch TV (Geospiza, Inc.) and MEGA 5.0 [27] were used to check electropherograms and align sequences with others obtained from GenBank.

## Results

A total of 4,760 females of sand flies belonging to ten genera and twenty-three species were tested (Table 1), and arranged in 1,289 pools (263 from peridomicile areas and 1,026 from the trails—Table 2).

**Table 1:** Females of sand flies collected during the study period in the Xakriabá Indigenous Reserve, Minas Gerais, Brazil.

Species	Collection sites		Total
	Peridomicile areas	Trails	
*Brumptomyia avellari*	3	2	5
*Evandromyia cortelezzii*	7	0	7
*Evandromyia lenti*	17	68	85
*Evandromyia* sp. *	0	4	4
*Evandromyia sallesi*	2	0	2
*Evandromyia spelunca*	5	322	327
*Evandromyia termitophila*	1	29	30
*Lutzomyia cavernicola*	0	1026	1026
*Lutzomyia ischnacantha*	10	220	230
*Lutzomyia longipalpis*	288	41	329
*Lutzomyia* sp. *	0	39	39
*Lutzomyia renei*	6	167	173
*Martinsmyia minasensis*	1	1235	1236
*Micropygomyia capixaba*	1	207	208
*Micropygomyia goiana*	14	269	283
*Micropygomyia longipennis*	0	22	22
*Micropygomyia peresi*	5	171	176
*Micropygomyia* sp. *	0	13	13
*Micropygomyia schreiberi*	0	47	47
*Migonemyia migonei*	4	3	7
*Nyssomyia intermedia*	361	139	500
*Nyssomyia neivai*	0	2	2
*Nyssomyia whitmani*	2	0	2
*Pintomyia misionensis*	0	1	1
*Pintomyia serrana*	2	1	3
*Psathyromyia* sp. *	0	1	1
*Scyopemyia sordellii*	0	2	2
**Total**	**729**	**4031**	**4760**

* Specimens had damaged morphological structures essential for identification.

**Table 2:** Sand flies collected and grouped in pools of up to ten specimens according to collection site within the Xakriabá Indigenous Reserve, Brazil.

Species	Sand flies pools from collection sites		Total
	Peridomicile	Trails	
*Brumptomyia avellari*	3	2	5
*Evandromyia cortelezzii*	6	-	6
*Evandromyia lenti*	15	40	55
*Evandromyia* sp. *	-	4	4
*Evandromyia sallesi*	1	-	1
*Evandromyia spelunca*	1	86	87
*Evandromyia termitophila*	1	20	21
*Lutzomyia cavernicola*	-	176	176
*Lutzomyia ischnacantha*	7	100	107
*Lutzomyia longipalpis*	111	28	139
*Lutzomyia* sp. *	-	1	1
*Lutzomyia renei*	6	40	46
*Martinsmyia minasensis*	1	170	171
*Micropygomyia capixaba*	1	45	46
*Micropygomyia goiana*	11	98	109
*Micropygomyia longipennis*	-	11	11
*Micropygomyia peresi*	5	80	85
*Micropygomyia* sp. *	-	13	13
*Micropygomyia schreiberi*	-	16	16
*Migonemyia migonei*	4	3	7
*Nyssomyia intermedia*	86	86	172
*Nyssomyia neivai*	-	2	2
*Nyssomyia whitmani*	2	-	2
*Pintomyia misionensis*	-	1	1
*Pintomyia serrana*	2	1	3
*Psathyromyia* sp. *	-	1	1
*Scyopemyia sordellii*	-	2	2
**Total**	**263**	**1026**	**1289**

* Specimens had damaged morphological structures essential for identification and so pools contained only a single specimen.

The ITS1-PCR did not detect the 300–350 bp fragment that characterizes the sample as positive for *Leishmania* in peridomicile samples. Thus, amplification using the ssu rDNA primers was performed as suggested by Schonian et al., [23]. The LnPCR detected *Leishmania* DNA in eleven samples (4.1%) belonging to *Ny*. *intermedia* (four), *Lu*. *renei* (two), *Lu*. *longipalpis* (two) and one sample of each following species: *Lu*. *ischnacantha*, *Mi*. *goiana* and *Ev*. *lenti*. DNA sequencing identified to the species level the *Leishmania* in nine out of eleven pools (81%) (results summarized in Table 3). The two pools (19%) for which species identification was not possible had hits for *Leishmania (Viannia)* sp. using the GenBank Blast tool. The most prevalent species of *Leishmania* in peridomicile areas was *Leishmania infantum chagasi* (36.3%), however when grouped the other species of *Leishmania* associated with ACL, the rate of natural infection was 63.6%.

**Table 3:** Pools of phlebotomine sand flies species naturally infected by *Leishmania* by collection site within the Xakriabá Indigenous Reserve, Brazil.

Species	Sand flies pools	*Leishmania* infection (infection rate)										Total
		*Le. braziliensis*		*Le. guyanensis*		*Le. (Viannia) sp.*		*Le. infantum*		*Le. amazonensis*		
		Peridomicile	Trails	Peridomicile	Trails	Peridomicile	Trails	Peridomicile	Trails	Peridomicile	Trails	
*Evandromyia lenti*	55	0	0	0	0	0	0	1	1	0	0	2 (8.6)
*Lutzomyia ischnacantha*	107	0	0	0	0	0	0	1	0	0	0	1 (4.3)
*Lutzomyia longipalpis*	139	0	0	0	0	1	0	1	0	0	0	2 (8.6)
*Lutzomyia renei*	46	0	0	1	0	1	0	0	0	0	0	2 (8.6)
*Martinsmyia minasensis*	171	0	1	0	3	0	0	0	0	0	1	5 (21.7)
*Micropygomyia capixaba*	46	0	1	0	0	0	0	0	0	0	0	1 (4.3)
*Micropygomyia goiana*	109	0	0	1	0	0	0	0	0	0	0	1 (4.3)
*Micropygomyia peresi*	85	0	1	0	0	0	0	0	1	0	0	2 (8.6)
*Nyssomyia intermedia*	172	2	2	0	0	0	0	1	1	1	0	7 (31)
Other species	359	0	0	0	0	0	0	0	0	0	0	0 (0)
Total	**1289**	**2 (28.5)**	**5 (71.5)**	**2 (40)**	**3 (60)**	**2 (100)**	**0 (0)**	**4 (57.1)**	**3 (42.9)**	**1 (50)**	**1 (50)**	**23 (100)**
		**7 (30.4)**		**5 (21.7)**		**2 (8.75)**		**7 (30.4)**		**2 (8.75)**		

**Fig 2:**
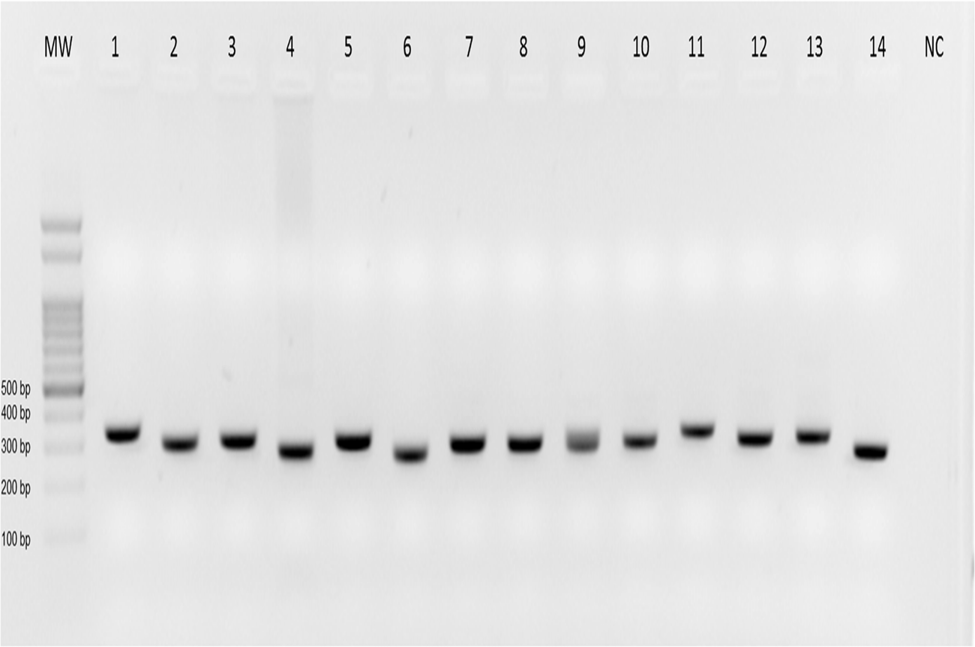
Detection of *Leishmania* in sand flies collected from trails in the Xakriabá Indigenous Reserve, Brazil, during the study period. Sand flies were pooled with up to ten specimens of the same locality, species and date, and ITS1 PCR was performed with total DNA extracted from these pools. The figure represents an ethidium bromide-stained 2% agarose gel in which the amplicons were submitted to electrophoresis. Lanes: MW, molecular weight marker—100 bp; lanes 1–4, positive controls of *Le*. *amazonensis* (IFLA/BR/67/PH8), *Le*. *braziliensis* (MHOM/BR/75/M2903), *Le*. *infantum* (MHOM/BR/74/PP75), *Le*. *guyanensis* (MHOM/BR/75/M4147) respectively; 5–14, phlebotomine positive pools; NC, negative control.

**Fig 3:**
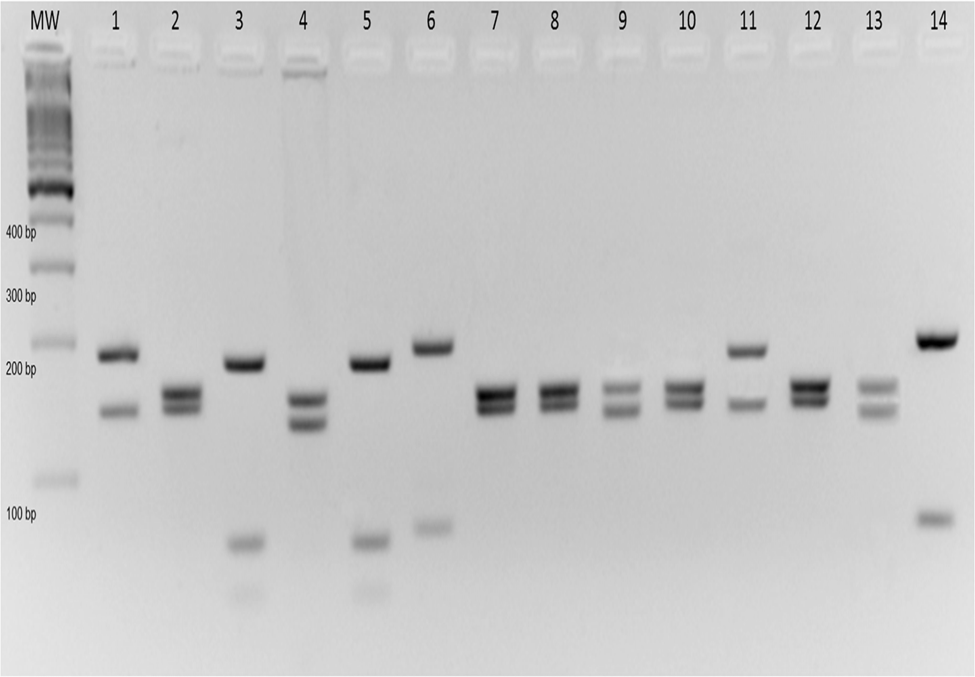
Species identification of *Leishmania* from sand flies collected from trails in the Xakriabá Indigenous Reserve, Brazil, during the study period. Sand flies were grouped in pools of up to ten specimens of the same species, locality, and date, and ITS1 PCR-RFLP was performed with total DNA extracted from these pools. The figure represents an ethidium bromide-stained 4% agarose gel in which the amplicons were submitted to electrophoresis. Lanes: MW, molecular weight marker—100 bp; lanes 1–4, positive controls of *Le*. *amazonensis* (IFLA/BR/67/PH8), *Le*. *braziliensis* (MHOM/BR/75/M2903), *Le*. *infantum* (MHOM/BR/74/PP75), *Le*. *guyanensis* (MHOM/BR/75/M4147) respectively; 5–14, phlebotomine positive pools.

Out of the 1,026 pools from the trails twelve were ITS1-PCR positive (Table 3). The twelve positive pools were *Mt*. *minasensis* (five), *Ny*. *intermedia* (three), *Mi*. *peresi* (two) and one sample of each of the following species: *Mi*. *capixaba* and *Ev*. *lenti* (Fig 2). The PCR-RFLP technique identified to the species level the *Leishmania* in all samples (Fig 3). The most prevalent species was *Leishmania braziliensis* (41.6%), followed by *Leishmania guyanensis* and *Leishmania infantum chagasi* (25% of each species).

## Discussion

One important step towards the incrimination of *Leishmania* vectors is the occurrence of naturally infected sand flies [28], however, other steps are also necessary for vector incrimination [4,28,29]. Although sand fly digestive tract dissection is the gold-standard method used to study the rate of natural infection in endemic areas, it is laborious and time consuming. Another limiting factor is the difficulty of processing a large number of samples that would be required for epidemiological investigations [30,31]. Moreover, in putative positive cases revealed by sand fly gut dissection, the infection has to be confirmed by in vitro culture of parasites (often susceptible to contamination), or by inoculation into laboratory animals, as other non-identified flagellates are commonly found in the insect midgut [32,33]. Alternatively, molecular techniques allow for DNA detection of a single *Leishmania* parasite [34] and probably represent a more sensitive tool than manual dissection and microscopic examination [35], which may underestimate natural sand fly infection rates in cases of low parasitaemia.

The use of PCR for detection of *Leishmania* DNA in wild sand flies is a useful technique for the identification of putative *Leishmania* vectors in different geographical areas [30,31,34,36,37]. The main advantages of molecular methods are their sensitivity and specificity, independent of the number, stage and location of the parasite in the insect midgut [38].

The present study collected a total of 4,760 specimens from the Xakriabá Indigenous Reserve, where recent ACL human cases and canine visceral leishmaniasis (VL) have been reported [20]. The species most frequently collected from peridomicile areas were *Ny*. *intermedia* and *Lu*. *longipalpis*, reinforcing the epidemiological role of these species in the transmission of *Leishmania braziliensis* and *Leishmania infantum chagasi*, respectively. The species most frequently collected from the trails was *Lu*. *cavernicola*, followed by *Mt*. *minasensis*; the epidemiological importance of both of these species remains unclear.

The natural infection rate found in peridomicile areas (4.1%) was higher than that observed among the trails (1.1%). This fact can be explained by the use of different molecular targets for PCR, since LnPCR (used only for the peridomicile pools) is more sensitive than ITS1-PCR [23]. Nonetheless, *Lu*. *longipalpis* and *Ny*. *intermedia*, both incriminated vectors of *Leishmania*, were found at high rates in peridomestic areas but represented only 1.4% and 3% of the total specimens collected, respectively, from the trails. It should be noted, however, that the peridomicile areas and the trails were sampled at different times, which can also influence detection of *Leishmania* in phlebotomine sand flies.

The finding of *Ev*. *lenti* associated with *Le*. *infantum chagasi* in both study areas (peridomicile and trails) is not consistent with that reported by Brazil et al. [39], who demonstrated the resistance of *Ev*. *lenti* collected in Minas Gerais to *Leishmania*. However, Margonari et al. [16] and Paiva et al. [40] have reported finding *Leishmania braziliensis* in these sand fly species using molecular techniques. In Campo Grande, Mato Grosso do Sul state, this species was closely associated with the peridomestic areas [41] and domestic animal shelters in rural areas [42], as it was observed in our study. Furthermore, Martins et al. [43], reported the significant association between this sand fly species and CL cases in the state of Goiás. Furthermore, Sherlock (1957) [44] and Sherlock & Miranda (1992) [45] reported finding natural infection by promastigotes on this sand fly species. Moreover, according Pinto et al (2012) [46], *Ev*. *lenti* and *Lu*. *longipalpis* seem to share the same ecological preferences (dry climate areas in different states of Brazil), and the presence of *Ev*. *lenti* justifies the establishment of epidemiological surveillance in the area to monitor the appearance of visceral leishmaniasis.

In this study, we observed three species of the genus *Lutzomyia* associated with *Leishmania* parasites: *Lutzomyia ischnacantha*, *Lutzomyia longipalpis* and *Lutzomyia renei*. The finding of *Leishmania* infection in species belonging to this genus is commonly reported in Brazil: Savani et al. [47] reported the infection of *Lutzomyia almerioi* by *Le*. *infantum chagasi* in Mato Grosso do Sul; Pita-Pereira et al. [48] and Missawa et al. [49] reported the infection in *Lutzomyia cruzi* and *Lutzomyia forattinii* by *Le*. *infantum chagasi* in the same state. However, reports of *Leishmania* infections in this genus are mostly related to *Lutzomyia longipalpis* and its association with *Leishmania infantum chagasi* [10,14,35]. In addition, Paiva et al. [40] reported the association of this sand fly species with *Le*. *braziliensis* and Savani et al. [47] with *Le*. *amazonensis*.

*Lutzomyia longipalpis* was found associated with *Leishmania infantum chagasi* and a parasite belonging to subgenus *Leishmania (Viannia)* sp.. These findings reinforce the reports about the epidemiological importance of this species mainly in the transmission of *Le*. *infantum chagasi* in Brazil [4,50,51,52]. The finding of this sand fly species with a parasite of the subgenus *Viannia* agrees with the findings of Paiva et al. [40]. However, this is not sufficient to incriminate this sand fly as a vector of species of *Leishmania* that cause ACL, despite the fact that several studies on experimental infections showed high susceptibility of *Lu*. *longipalpis* to different *Leishmania* species [53,54,55].

*Lutzomyia renei* and *Lutzomyia ischnacantha* were never found with *Leishmania* parasites. It is known that the species of this genus are mostly attracted to a diversity of hosts and use a variety of habitats [56]. The finding of *Lu*. *renei* with *Leishmania guyanensis* and *Leishmania (Viannia)* sp. DNA, and *Lutzomyia ischanacantha* with *Le*. *infantum chagasi* DNA in XIR may be occasional and with no epidemiological importance or it might be associated with transmission of *Leishmania* to wild and synanthropic hosts as reported by Quaresma et al. [20] in a study conducted in the same area. To define the role of these sand fly species in the epidemiological context of leishmaniasis, additional studies are necessary.

*Martinsmyia minasensis* is a phlebotomine species whose feeding habits may be closely related to rodents in the study area [21]. This species was found associated with three species of the genus *Leishmania*; *Le*. *guyanensis* was the most common (3/5 positive pools), followed by *Le*. *braziliensis* and *Le*. *amazonensis* in the same proportions (1/5 positive pools). The finding of *Leishmania guyanensis* in the same study area was reported by Quaresma et al. [20] in *Thrichomys apereoides* (Rodentia: Echimyidae) and *Marmosops incanus* (Didelphimorphia: Didelphidae). The ecological role of this species should be studied in order to elucidate the epidemiological role in the wild and peridomestic *Leishmania* transmission cycles. The finding of *Mt*. *minasensis* infected by *Leishmania braziliensis* can also be related to the finding of this parasite in rodents in the same area of study [20], although the finding of *Le*. *amazonensis* in the XIR has never been previously reported and human infection by this parasite species is not common, even though it has been identified in some regions of Brazil [57,58,59,60,61,62,63]. The main vector of *Le*. *amazonensis* in northern Brazil, *Bichromomyia flaviscutellata* [64,65,66,67], was not recorded by us during the study period.

The finding of *Leishmania* in the genus *Micropygomyia* does not correspond with the behavioral habits of this group, since they have been reported to feed on cold-blooded animals whose participation in the cycle of leishmaniasis is not known in Brazil [56,68,69]. Natural infection with flagellates has been reported in Venezuela in *Mi*. *atroclavata* [70,71,72], *Mi venezuelensis* [72] and *Mi cayennensis* [71]. Deane et al. [73] also reported infections with flagellates in *Mi*. *cayennensis* captured near a bat cave in Venezuela. In Brazil, *Micropygomyia ferreirana* and *Micropygomyia quinquefer* have been reported associated with *Leishmania braziliensis* through molecular methods in the states of Espírito Santo [74] and Mato Grosso [40]. The finding of *Leishmania* DNA in this genus assumes that these sand flies have fed on hosts susceptible to parasite infection, which is unclear and unknown in cold-blooded animals in Brazil.

*Nyssomyia intermedia* were positive for *Le*. *braziliensis* in four samples (two from each area) reinforcing the epidemiological role of these species in the transmission cycle of ACL given its high abundance in endemic areas in Minas Gerais [75]. It is noteworthy that this species was predominant in peridomestic areas, corroborating the findings of Gontijo et al. [8] in Vale do Jequitinhonha, Minas Gerais and Saraiva et al., [76] in same state, where this species was abundant in environments with anthropic modification.

In this study *Ny*. *intermedia* was found associated with *Le*. *infantum chagasi* both in peridomicile areas and among the trails, however, the role of this sand fly species in the epidemiological cycle of this parasite is unclear. Oliveira et al. [77] related in a study conducted in VL focus in an indigenous village in Minas Gerais, a high incidence of this species in the absence of *Lu*. *longipalpis*, and the same results was reported by Coelho et al. [78] in a VL focus in Goiás state. In addition, *Ny*. *intermedia* has been experimentally infected with *Le*. *infantum chagasi* [79,80] and recently was found associated with this species of *Leishmania* in Belo Horizonte, MG [14]. The significance of finding this phlebotomine with *Le*. *amazonensis* in the present study is unclear, but in an experimental study Paiva et al., [81] reported this sand fly infected with this parasite. Despite this fact, little is known about the vectorial capacity of this sand fly for this parasite in natural environments.

The entomological data reported in this study may be closely related to the environment found in the XIR: a transition area between cerrado and caatinga biomes, with anthropic modified areas (peridomicile) near forested areas and a variety of wild, synanthropic and domestic animals potentially involved in the transmission cycle of *Leishmania* [20]. The finding of phlebotomine vectors, as well as species that have no known epidemiological role associated with *Leishmania* species, reinforces the heterogenous nature of the study area, and calls for additional studies to investigate the vectorial capacity of these species.
